# Exposure to fogger trucks and breast cancer incidence in the Long Island Breast Cancer Study Project: a case–control study

**DOI:** 10.1186/1476-069X-12-24

**Published:** 2013-03-15

**Authors:** Alexandra J White, Susan L Teitelbaum, Mary S Wolff, Steven D Stellman, Alfred I Neugut, Marilie D Gammon

**Affiliations:** 1 Department of Epidemiology, University of North Carolina, Chapel Hill, North Carolina, USA; 2 Department of Preventive Medicine, Mount Sinai School of Medicine, New York, New York, USA; 3 Department of Epidemiology, Columbia University, New York, New York, USA; 4 Department of Medicine, Columbia University, New York, New York, USA

**Keywords:** Organochlorines, Dichlorodiphenyltrichloroethane (*p,p’*-DDT), 1,2,-Dichloro-2,2-bis (p-chlorophenyl)ethylene (DDE), Pesticides, Cancer, Breast

## Abstract

**Background:**

Few studies have supported an association between breast cancer and DDT, usually assessed with biomarkers that cannot discern timing of exposure, or differentiate between the accumulation of chronic low-dose versus acute high-dose exposures in the past. Previous studies suggest that an association may be evident only among women exposed to DDT during biologically susceptible windows, or among those diagnosed with estrogen receptor/progesterone receptor-positive (ER+PR+) breast cancer subtypes. Self-reported acute exposure to a fogger truck, which sprayed DDT prior to 1972, was hypothesized to increase the risk of breast cancer, particularly among women exposed at a young age or diagnosed with ER+PR+ breast cancer.

**Methods:**

We examined these possibilities in the Long Island Breast Cancer Study Project (LIBCSP) (1,508 cases, 1,556 controls), which included exposure assessment by structured questionnaire and serum samples collected between 1996–1998, using adjusted logistic and polytomous regression to estimate ORs and 95% CIs.

**Results:**

Women with ER+PR+ breast cancer had a 44% increased odds of ever seeing a pre-1972 fogger truck compared to other subtypes (OR = 1.44; 95% CI 1.08-1.93). However, there was little variation in the observed increase in breast cancer risk when considering all women who reported seeing a pre-1972 fogger truck at their residence (OR = 1.16; 95% CI 0.98, 1.37), or during hypothesized susceptible windows. Self-reported acute exposure was not correlated with serum concentrations, a biomarker of long-term exposure.

**Conclusions:**

These findings support the hypothesis that seeing a fogger truck, a proxy measure for acute DDT exposure, may be associated with ER+PR+ tumors, the most commonly diagnosed breast cancer subtype among American women.

## Background

Breast cancer is the leading cancer diagnosis among women in the United States (U.S.) [1]. There is substantial evidence that the etiology of breast cancer may differ by receptor-defined subtypes [2]. The most commonly diagnosed subtypes among American women are luminal A and luminal B tumors [3], which together can be defined as estrogen and progesterone receptor positive (ER+PR+) tumors [4,5], and are often associated with estrogen-related risk factors such as early age at menarche and nulliparity [6-8].

**Figure 1:**
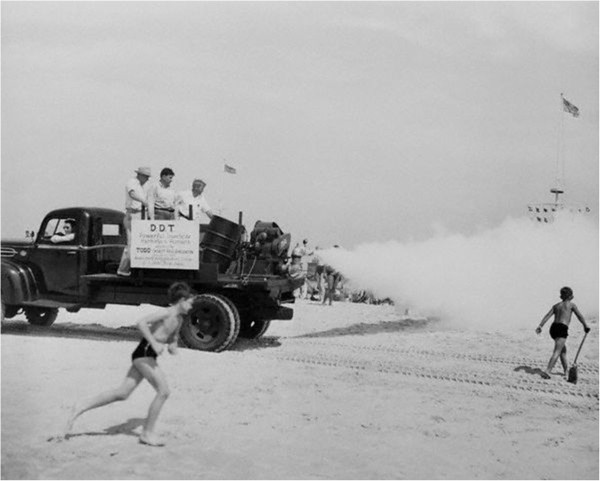
Fogger truck sprays Jones Beach in New York with DDT, 1945 (source: Corbis Images).

**Figure 2:**
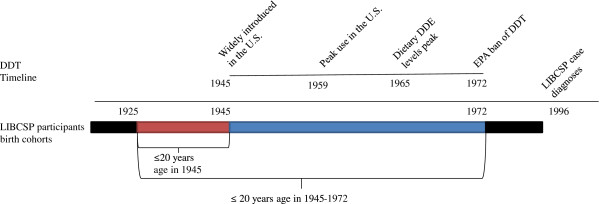
Timeline of DDT use in the United States and birth cohorts of LIBCSP participants, LIBCSP, 1996–1997.

There has long been suspicion of a relationship between the persistent organochlorine pesticide *p,p’-*dichlorodiphenyltrichloroethane (*p,p’*-DDT) and breast cancer, although results have been inconsistent [9]. By 1945, *p,p’*-DDT and its most prominent metabolite, *p,p’-*dichlorodiphenyldichloroethylene (*p,p’*-DDE), were widespread in the U.S. [10]. Fogger trucks sprayed DDT, the commercial product, on Long Island (Figure 1), and elsewhere across the nation, for the control of gypsy moths and mosquitoes until use greatly declined after being banned in 1972 (see timeline, Figure 2) [10]. *p,p’-*DDT exposure has been inconsistently linked to breast cancer despite having both estrogenic and carcinogenic properties [11,12]. Acute, high dose exposure to commercial DDT (which contains the more strongly estrogenic compounds *o’p-*DDT and *p’p-*DDT) has been shown to increase mammary cell proliferation in animal models [13,14] whereas chronic low-dose exposure through the diet is more predominately composed of the metabolite *p,p’*-DDE, which has little to no estrogenic effect [14]. DDT is still utilized to combat malaria, and more evidence regarding its role in possible health outcomes could either expand its use for vector control or encourage the use of other pesticides [15].

Early studies suggested that *p,p’-*DDE concentrations were associated with an increased risk of breast cancer [14,16]. However, most studies have supported little if any association [17-23]. Few studies have measured concentrations during developmental periods [24], which could represent a biologically plausible window of exposure for breast cancer risk [25,26], or among breast cancer subtypes. Instead, previous investigations often relied on biomarker assessments of *p,p’-*DDT metabolites. Positive correlations are observed with age and are likely due to the relatively long half-lives (more than 4 years for *p,p’-*DDT [27] and 10 years for *p,p’-*DDE [28]). Whether measured in fat or blood specimens, these biomarkers are therefore more likely to reflect cumulative, lifetime exposure. Thus, exposures during specific time periods, or acute versus chronic exposures, are not readily discernible, especially when these long-term biomarkers are assessed in blood samples collected long after the critical exposure of interest. Between 2000 and 2001, Cohn and colleagues [24] measured *p,p’-*DDT concentrations in serum samples collected beginning in 1959, the year of peak DDT use, and ending in 1967, when DDT was still in use. Among women who were younger than 14 in 1945, those with blood concentrations in the highest tertile were five times more likely to develop breast cancer compared to the lowest tertile. This is consistent with the hypothesis that breast cancer risk can be affected by events early in life [29], and there is interest at a national level regarding exposures that occur during adolescence and subsequent breast cancer risk [25].

The aim of this study was to investigate the relationship between self-reported exposure to a fogger truck at a residence prior to the ban of DDT, as a proxy measure of an acute high dose exposure, and breast cancer incidence, particularly among women diagnosed with ER+PR+ tumors. In addition, the association between adolescent exposure to fogger trucks during two demonstrated developmentally sensitive time periods, defined as ≤20 years (consistent with John and Kelsey, 1993 [30]) and less ≤14 years (consistent with Cohn *et al.,* 2007 [24]), and breast cancer risk was determined. Further, the relationship between fogger truck exposure and breast cancer risk was assessed by birth cohort.

## Methods

Data for this case–control investigation were drawn from the Long Island Breast Cancer Study Project (LIBCSP), which is described in detail in Gammon *et al.*[31] Briefly, LIBCSP is a population-based study of breast cancer focusing on female residents in Nassau and Suffolk counties in Long Island, New York. IRB approval was obtained from all participating institutions.

### Study population

All participants were residents of Nassau or Suffolk counties at the time of diagnosis and spoke English. Cases were women newly diagnosed with a first primary *in situ* or invasive breast cancer between August 1st, 1996 and July 31st, 1997, confirmed by a physician or the medical record. Cases were ascertained by contacting pathology departments of all 28 hospitals on Long Island and three tertiary care hospitals in New York City.

Controls were randomly selected women who had no history of breast cancer and were frequency matched to cases based on the expected age distribution of case women by 5-year age groups. Potential controls were identified by random digit dialing for those less than 65 years of age and by Health Care Finance Administration rosters for those 65 years and older.

Written signed informed consent was obtained from all participants prior to interview. A total of 1,508 cases and 1,556 controls (82.1% and 62.7%, respectively, of all eligible subjects) completed the interview process. Participants were women aged from 20 to 98 years, and most likely to be postmenopausal (67%); in addition, the overwhelming majority of women self-identified as white (93%), rather than as black (5%), which is consistent with the racial distribution of these two counties at the time of data collection [31].

Cases (97.7%) signed a medical record release form for the abstraction of clinical characteristics of breast cancer diagnosis and final study eligibility determination. For 95.2% of cases, medical records were successfully located and abstracted.

### Assessment of fogger truck exposure

Exposure to fogger trucks was assessed by questionnaire. The LIBCSP case–control interview occurred in the respondents’ homes and, for cases, within several months of the diagnosis of the first primary breast cancer. All participants were administered the main case–control questionnaire by uniformly trained interviewers. The main questionnaire included a comprehensive assessment of known and suspected risk factors for breast cancer, including a section on environmental exposures. This section included a question asking whether a fogger truck was seen at a participant’s residence and if yes the ages or the dates at which they began and ceased living at the residence. Study participants were able to list 2 residences where they witnessed a fogger truck. Any other exposures to fogger trucks at non-residences would not be included in this question. Participants were also asked whether they chased a fogger truck during childhood or adolescence; however, due to low levels of a positive response that question was not included in this analysis.

To maximize information regarding windows of exposure, we utilized a birth cohort approach (see Figure 2). Specifically, the relationship between fogger truck exposure and breast cancer risk was assessed across 4 birth cohorts (<1925, 1925–1945, 1946–1972, >1972). Exposure at ≤20 years of age was defined as the hypothesized susceptible period, a time that encompasses both puberty and time before first birth for 90% of the LIBCSP population. The first (<1925) and last (>1972) birth cohorts would not have been exposed to DDT when ≤20 years of age. The birth cohort born between 1925 and 1945 would have been ≤20 years of age at the time of the introduction of DDT in 1945 [10] and those born in 1946–1972 would have been ≤20 years of age when DDT exposure was ubiquitous.

### Other covariate assessment

Other LIBCSP data used for the current study include information collected as part of the in-person interview, from serum, or case medical record abstraction, as described below.

Responses to other sections of the main questionnaire, including reproductive and menstrual histories, life course body size, family history of breast cancer and race, were used when considering potential confounders and/or effect modifiers. Distributions of known risk factors among the study population have been described in full in Gammon *et al.*[31]. Serum were used to assess serum *p,p’-*DDT and *p,p’-*DDE concentrations. Certified phlebotomists or nurses obtained blood samples from 73.1% and 73.3% of cases and controls, respectively. Blood samples were randomly selected from participants who donated blood between 1996 and 1998, with a blood volume of greater than 1.5 ml, from all African-Americans and from all cases with *in situ* breast cancer [17]. The Brock *et al.*[32] method was used to determine serum concentrations of *p,p'*-DDE and *p,p’*-DDT. Limits of detection were about 0.2 ng/ ml for both *p,p’*-DDT and *p,p’*-DDE. *p,p’*-DDE serum concentrations were attained for 643 cases and 427 controls and *p,p’*-DDT serum concentrations were attained for 633 cases and 418 controls [17].

Medical records of the cases were abstracted for 1,402 women to obtain data on hormone receptor status of the first primary breast cancer [17]. These data were used to classify cases by breast cancer subtypes. Information on hormone receptor status was available from the medical record for 990 cases (65.6%). As previously reported [17], among the LIBCSP breast cancer cases, the first primary tumor was ER+PR+ for 583 cases (58.9%), ER-PR- for 212 (21.4%), ER-PR+ for 52 (5.3%), and ER+PR- for 143 (14.4%).

### Statistical methods

Descriptive statistics were determined for covariates, stratified by birth cohort and case status. Unconditional logistic regression [33] was used to estimate odds ratios (ORs) and 95% confidence intervals (CIs) for associations between breast cancer and reporting a fogger truck at a residence. The exposure was further stratified by whether the woman reported seeing a fogger truck ≤1972; if a woman reported seeing a fogger truck in both time periods, she was categorized in the ≤1972 group. Less than 10% of cases (n = 122) and controls (n = 124) reported seeing a fogger truck after the ban on DDT. Further analyses were restricted to those women who reported seeing a fogger truck ≤1972 and those who never reported seeing a fogger truck (n =1365 cases and 1406 controls); estimates for the total LIBCSP population are also shown. There were women missing information on which residence they saw a fogger truck (n = 26 controls and n = 21 cases) and 4 women without sufficient information on ages of exposure to be classified who are not included in the sub analyses. To explore for possible age periods of increased susceptibility, two windows were considered: ≤14 and ≤20 years of age. In doing so, the exposed group was restricted to those women who reported seeing a fogger truck at the residence they began living ≤14 and ≤20 years of age, respectively. The association between the number of residences a participant reported seeing a fogger truck (defined as 0, 1 and 2) and breast cancer risk was determined. Disjoint categories of age were also investigated (≤14, 15–20, >20 years of age).

All statistical models were adjusted for the frequency matching factor, 5-year age group. Other potential confounders were identified (through a thorough review of the relevant literature and the use and analysis of a directed acyclic graph (DAG) [34]), and include: change in weight from age 20 to diagnosis (lost, stable, gained), menopausal status (pre, post), breastfeeding history (ever, never), family history of breast cancer in mother, sister or daughter (yes, no) and race (white, black, other). Confounding was evaluated by these covariates using backward selection from the full model identified by our causal diagram with a 10% change in estimate criterion. None of the covariates changed the estimate more than 10%, and thus only age group was included in the final models.

Polytomous logistic regression [35] was used to estimate the ratio of the ORs and 95% CI for associations between ever seeing a fogger truck at a residence when comparing ORs for breast cancer subtypes, as defined by combined ER/PR status (ER+PR+ vs. all other subtypes, and ER-PR- vs. all other subtypes). The associations between seeing a fogger truck during possibly susceptible periods, ≤14 years of age and ≤20 years of age, and breast cancer subtypes was also investigated.

Effect measure modification on a multiplicative scale was assessed by comparing the multivariable models with and without cross-product terms of the exposure and weight change [35]. To determine modification by weight gain, participants were stratified by whether they lost weight, maintained their weight within 3% in lbs [36], or gained weight from age twenty to age at reference (which was the date of the first primary breast cancer diagnosis for cases and the date of study identification for controls). Other cutpoints were considered, including a 10 kg change in weight (data not shown); the cutpoints used here were believed to best represent the data. Weight gain as a dichotomous variable (with stable weight plus weight loss as the referent) was also considered as a possible effect modifier, but no significant interaction was noted (data not shown). Effect measure modification was also evaluated for breastfeeding status (ever, never) and menopausal status (pre, post).

The analysis was further stratified by birth cohort, based on important years in the history of DDT use [10]. The association between seeing a fogger truck at a residence and breast cancer within each birth cohort was evaluated (<1925, 1925–1945, 1946–1972, >1972).

To assess correlation with serum concentrations and self-report of a fogger truck at a residence, a kappa statistic [37] was calculated with lipid-adjusted *p,p’-*DDT and *p,p’-*DDE concentrations were dichotomized at the median. Linear regression was also employed to test whether self-report of a fogger truck predicted log-transformed *p,p’-*DDT or *p,p’-*DDE concentrations or the *p,p’-*DDT/ *p,p’-*DDE ratio in the serum with adjustment for lipid concentrations [38], 5-year age groups, triglycerides, cholesterol, change in weight from age 20 to diagnosis, current BMI, breastfeeding history and race [33]. An interaction between current BMI and change in weight was investigated (data not shown) but was not a strong predictor and did not remain in the models. Positive and zero values of individual organochlorine concentrations below detection limit was set to the lowest positive value for that compound observed in samples.

All analyses were completed using SAS 9.2 (Cary, NC).

## Results

The majority of the LIBCSP study population was born in the two birth cohorts that were exposed to DDT at young ages, 1925–1945 and 1946–1972 (Table 1). As would be expected, lactation history and menopausal status vary by birth cohort.

**Table 1:** Descriptive characteristics by birth cohort, LIBCSP, 1996-1997

		**Birth cohorts**^**a**^					
		**<1925**		**1925-1945**		**1946-1972**	
		**N = 465**		**N = 1,587**		**N = 1,008**	
		**Cases**	**Controls**	**Cases**	**Controls**	**Cases**	**Controls**
		**N (%)**	**N (%)**	**N (%)**	**N (%)**	**N (%)**	**N (%)**
Age at reference	<35	0 (0.0)	0 (0.0)	0 (0.0)	0 (0.0)	39 (8.4)	41 (7.5)
	35-44	0 (0.0)	0 (0.0)	0 (0.0)	0 (0.0)	181 (39.1)	245 (45.0)
	45-54	0 (0.0)	0 (0.0)	154 (19.7)	164 (20.3)	243 (52.5)	259 (47.5)
	55-64	0 (0.0)	0 (0.0)	372 (47.7)	403 (49.9)	0 (0.0)	0 (0.0)
	65-74	111 (41.9)	70 (35.0)	254 (32.6)	240 (29.7)	0 (0.0)	0 (0.0)
	75-84	134 (50.6)	112 (56.0)	0 (0.0)	0 (0.0)	0 (0.0)	0 (0.0)
	85+	20 (7.5)	18 (9.0)	0 (0.0)	0 (0.0)	0 (0.0)	0 (0.0)
Household Income	<20,000	95 (36.0)	63 (31.7)	74 (9.5)	77 (9.6)	19 (4.1)	28 (5.1)
	20,000-34,999	112 (42.4)	82 (41.2)	195 (25.1)	193 (23.9)	36 (7.8)	62 (11.4)
	35,000-49,999	28 (10.6)	23 (11.6)	159 (20.5)	157 (19.5)	56 (12.1)	83 (15.2)
	50,000-69,999	16 (6.1)	14 (7.0)	121 (15.6)	154 (19.1)	115 (24.8)	120 (22.0)
	70,000-89,000	7 (2.7)	7 (3.5)	91 (11.7)	105 (13.0)	92 (19.9)	96 (17.6)
	>90,000	6 (2.3)	10 (5.0)	137 (17.6)	120 (14.9)	145 (31.3)	156 (28.6)
Body Mass Index at referent	<18.5	<5^b^ (1.5)	9 (1.1)	8 (1.0)	9 (1.1)	15 (3.2)	9 (1.7)
	18.5-24.9	99 (38.2)	337 (42.4)	282 (36.7)	337 (42.4)	262 (56.6)	285 (53.1)
	25.0-29.9	97 (37.5)	248 (31.2)	270 (35.1)	248 (31.2)	116 (25.1)	144 (26.8)
	>30	59 (22.8)	201 (25.3)	209 (27.2)	201 (25.3)	70 (15.1)	99 (18.4)
Lactation	Never	173 (65.3)	114 (57.0)	571 (73.2)	569 (70.5)	284 (61.3)	316 (58.0)
	Ever	92 (34.7)	86 (43.0)	209 (26.8)	238 (29.5)	179 (38.7)	229 (42.0)
Menopausal Status	Pre-	0 (0.0)	0 (0.0)	75 (9.8)	56 (7.2)	397 (88.0)	443 (87.5)
	Post-	265 (100.0)	200 (100.0)	687 (90.2)	727 (92.8)	54 (12.0)	63 (12.5)

^a^ Participants born after 1972 (n<5, all controls) were not displayed for confidentiality purposes.

^b^ Cell is defined as <5 for confidentiality purposes.

In the LIBCSP population, 589 (39.1%) and 558 (35.9%) cases and controls, respectively, reported ever seeing a fogger truck at a personal residence on Long Island. When restricted to those who lived at their residences prior to the DDT ban in 1972, 446 (32.7%) and 408 (29.0%) cases and controls, respectively, reported seeing a fogger truck. As shown in Table 2, ever seeing a fogger truck at a residence was associated with little to no increase in risk of breast cancer (OR = 1.14; 95% CI: 0.98, 1.32) in the entire LIBCSP population. When the sample was restricted to the subset of women who lived at their residence ≤1972, seeing a fogger truck at a residence was similarly associated with a little to no increase in risk of breast cancer (OR = 1.16; 95% CI: 0.98, 1.37). Reporting seeing a fogger truck ever at a residence >1972 was not associated with breast cancer risk (OR = 1.07; 95% CI: 0.94, 1.23), with 11.7% (n = 122) and 11.1% (n = 124) of cases and controls, respectively, reporting seeing a fogger truck after the DDT ban. Unless otherwise noted, all of the results presented here will be for those who reported seeing a fogger truck ≤1972.

**Table 2:** **Association between fogger truck and breast cancer, stratified by time period and age at residence**^**a**^

**Population**	**Self-reported fogger truck at residence**	**Cases N (%)**	**Controls N (%)**	**Age-adjusted**
				**OR (95% CI)**^**b**^
Total LIBCSP population	Never	919 (60.9)	998 (64.1)	1.00 (reference)
	Ever	589 (39.1)	558 (35.9)	1.14 (0.98, 1.32)
	≤20 years old	161 (10.7)	168 (10.8)	1.04 (0.92, 1.18)
	≤14 years old	135 (9.0)	155 (10.0)	0.99 (0.88, 1.13)
Fogger truck ≤ 1972	Never	919 (67.3)	998 (71.0)	1.00 (reference)
	Ever	446 (32.7)	408 (29.0)	1.16 (0.98, 1.37)
	≤20 years old	159 (11.6)	166 (11.8)	1.08 (0.84, 1.37)
	≤14 years old	135 (9.9)	153 (10.9)	0.99 (0.76, 1.28)
Fogger truck > 1972^c^	Never	919 (88.3)	998 (88.9)	1.00 (reference)
	Ever	122 (11.7)	124 (11.1)	1.07 (0.94, 1.23)

^a^ Earliest self-reported fogger truck at residence among Long Island, New York women, Long Island Breast Cancer Study Project,1996-1997. “Total LIBCSP population” includes both fogger truck exposures ≤ 1972 and >1972, “Fogger truck <1972” and “Fogger truck >1972” definitions are mutually exclusive categories. “Ever” includes exposure >20, ≤20 and ≤14 years of age; “≤20 years of age” includes exposures ≤14 years of age.

^b^adjusted for 5-year age groups.

^c^Participants exposed (n<5) during susceptible windows not displayed for confidentiality purposes.

In subgroup analyses, 11.6% of cases and 11.8% of controls were found to be at or under the age of 20 when living at the residence where they saw the fogger truck. Slightly fewer, 9.9% of cases and 10.9% of controls were living at the residence ≤14 years of age. The associations between fogger truck and breast cancer were not stronger when limiting the exposed to participants who reported seeing a fogger truck at a residence that they lived ≤14 years of age (OR = 0.99; 95% CI: 0.76, 1.28) and ≤20 years of age (OR = 1.08; 95% CI: 0.84, 1.37) (Table 2).

There was little to no increase in risk for reporting seeing a fogger truck at 2 residences (OR = 1.15; 95% CI: 0.82, 1.61) (Table 3). When using disjoint categories for age at residence, there were positive but imprecise associations was for women who were 15–20 at the residence they saw a fogger truck (OR = 1.86; 95% CI: 0.98, 3.52). Due to limited sample sizes in these categories, the remaining subgroup analysis focuses on women who were ≤14 and ≤20 years of age at the reported residence.

**Table 3:** **Fogger truck and risk of breast cancer by number of residences and age at residence**^**a**^

**Population**	**Residential information**	**Self-reported fogger truck at residence**	**Cases**	**Controls**	**Age-adjusted**
			**N (%)**	**N (%)**	**OR (95% CI)**^**b**^
Total LIBCSP population	# of reported residences	0	919 (60.9)	998( 64.1)	1.00 (reference)
		1	459 (30.4)	423( 27.2)	1.16 (0.99, 1.37)
		2	130 (8.6)	135 (8.7)	1.07 (0.83, 1.40)
	Age at residence	None	919 (60.1)	998 (63.5)	1.00 (reference)
		>20 years old	445 (29.1)	403 (25.7)	0.94 (0.72, 1.21)
		15-20 years old	29 (1.9)	15 (1.0)	2.03 (1.08, 3.81)
		≤14 years old	135 (8.8)	155 (9.9)	0.94 (0.72, 1.21)
Fogger truck ≤1972	# of reported residences	0	919 (68.9)	998 (69.4)	1.00 (reference)
		1	335 (25.1)	367 (25.6)	1.16 (0.97, 1.38)
		2	79 (5.9)	73 (5.1)	1.15 (0.82, 1.61)
	Age at residence	None	919 (64.7)	998 (68.2)	1.00 (reference)
		>20 years old	339 (23.9)	297 (20.3)	1.19 (0.99, 1.43)
		15-20 years old	27 (1.9)	15 (1.0)	1.86 (0.98, 3.52)
		≤14 years old	135 (9.5)	153 (10.4)	0.92 (0.72, 1.21)

^a^ Self-reported fogger trucks at residence among Long Island, New York women, Long Island Breast Cancer Study Project,1996-1997, stratified by time period. Population definitions and exposure information are mutually exclusive categories.

^b^adjusted for 5-year age groups.

As shown in Table 4, women with ER+PR+ tumors had a significantly increased odds of ever seeing a fogger truck when compared to all other subtypes (OR = 1.44; 95% CI: 1.08, 1.93) when estimated as the ratio of the odds ratio using polytomous regression. In contrast, women who reported ever seeing a fogger truck did not have increased odds of having ER-PR- breast cancer compared to all other subtypes (OR = 0.91; 95% CI: 0.64, 1.29). When using a case-case analysis, comparing ER+PR+ to all other subtypes the OR was also significant (OR = 1.45; 95% CI: 1.10, 1.92). A pronounced, although imprecise, relationship with ER+PR+ tumors was observed for those who saw a fogger truck at a residence they lived at ≤20 (OR = 1.50; 95% CI: 0.97, 2.32) and ≤14 (OR = 1.15; 95% CI: 0.99, 1.33) years of age. Breast cancer tumor subtypes were also stratified by birth cohort, although study power was limited (see Additional file 1).

**Table 4:** **Associations between reporting a fogger truck and breast cancer subtype**^**a**^

**Population**	**Self-reported fogger truck at residence**	**Subtype**^**b**^	**Cases (N)**	**Controls (N)**	**Age-adjusted**^**c **^**ratio of the ORs (95% CI)**
Total LIBCSP population	Ever	Other subtypes	778	1,556	1.00 (reference)
		ER-PR-	212	1,556	0.98 (0.71, 1.35)
		Other subtypes	407	1,556	1.00 (reference)
		ER+PR+	583	1,556	1.41 (1.08, 1.84)
	≤20 years of age	Other subtypes	539	1,556	1.00 (reference)
		ER-PR-	152	1,556	0.91 (0.71, 1.16)
		Other subtypes	298	1,556	1.00 (reference)
		ER+PR+	393	1,556	1.25 (1.01, 1.54)
	≤14 years of age	Other subtypes	526	1,556	1.00 (reference)
		ER-PR-	146	1,556	0.84 (0.64, 1.11)
		Other subtypes	289	1,556	1.00 (reference)
		ER+PR+	383	1,556	1.32 (1.05, 1.67)
Fogger truck ≤1972	Ever	Other subtypes	708	1,530	1.00 (reference)
		ER-PR-	190	1,530	0.91 (0.64, 1.29)
		Other subtypes	373	1,530	1.00 (reference)
		ER+PR+	525	1,530	1.44 (1.08, 1.93)
	≤20 years of age	Other subtypes	538	1,528	1.00 (reference)
		ER-PR-	151	1,528	0.89 (0.53, 1.49)
		Other subtypes	297	1,528	1.00 (reference)
		ER+PR+	392	1,528	1.50 (0.97, 2.32)
	≤14 years of age	Other subtypes	526	1,528	1.00 (reference)
		ER-PR-	146	1,528	1.04 (0.87, 1.24)
		Other subtypes	289	1,528	1.00 (reference)
		ER+PR+	383	1,528	1.15 (0.99, 1.33)

^a^ Earliest self-reported fogger truck at residence stratified by time period and age at residence among Long Island, New York women, Long Island Breast Cancer Study Project,1996-1997. Populations are mutually exclusive categories; “Ever” includes exposure >20, ≤20 and ≤14 years of age; “≤20 years of age” includes exposures ≤14 years of age.

^b^ For ER+PR+ comparison, “other subtypes” includes ER-PR-, ER+PR-, ER-PR+. For ER-PR- comparison, “other subtypes” includes ER+PR+, ER+PR-, ER-PR+.

^c^ adjusted for 5-year age groups.

The covariate distributions for women with known ER/PR status were very similar to women without known ER/PR status (data not shown). However, the effect estimate for the association for ever seeing a fogger truck and breast cancer was slightly higher among case women with known ER/PR status (OR = 1.33; 95% CI: 1.11, 1.59) than among all women regardless of ER/PR status (OR = 1.16; 95% CI: 0.98, 1.37), although with overlapping confidence intervals.

The OR for the association between exposure to a fogger truck and breast cancer was explored for effect measure modification by adult weight change, breastfeeding history and menopausal status at diagnosis as shown in Table 5. Although weight change was not a statistically significant modifier of the relationship between fogger trucks and breast cancer (LRT *x*^*2*^ = 1.86, df = 1, p = 0.2), there was a slightly elevated association between women who gained weight and reported ever seeing a fogger truck at a residence compared to women who had gained weight but did not report seeing a fogger truck (OR = 1.21; 95% CI: 1.01, 1.45). Breastfeeding history and menopausal status were not found to be significant modifiers of the relationship between fogger trucks and breast cancer. However, a positive association for reporting a fogger truck and breast cancer risk was observed for postmenopausal women (OR = 1.24; 95% CI: 1.02, 1.51).

**Table 5:** **Fogger trucks and risk of breast cancer by weight change, breastfeeding and menopausal status**^**a**^

**Population**		**Modifiers**	**No fogger truck**		**Fogger truck**		**Age-adjusted OR (95% CI)**^**b**^		**p-value**^c^
			**Cases (N)**	**Controls (N)**	**Cases (N)**	**Controls (N)**	**No fogger truck**	**Fogger truck**	
Total population	Weight change	Lost	78	109	29	42	1.00 (reference)	0.94 (0.53, 1.68)	0.3
		Stable	80	75	32	34	1.00 (reference)	0.73 (0.39, 1.39)	
		Gain	814	761	528	482	1.00 (reference)	1.19 (1.01, 1.40)	
	Breastfeeding status	Never	612	633	416	370	1.00 (reference)	1.17 (0.97, 1.40)	0.7
		Ever	307	365	173	188	1.00 (reference)	1.08 (0.83, 1.40)	
	Menopausal status	Pre-	308	328	164	175	1.00 (reference)	0.96 (0.73, 1.26)	0.2
		Post-	592	633	414	357	1.00 (reference)	1.25 (1.05, 1.50)	
Fogger truck ≤1972	Weight change	Lost	78	109	23	32	1.00 (reference)	1.03 (0.54, 1.96)	0.2
		Stable	80	75	20	24	1.00 (reference)	0.68 (0.32, 1.43)	
		Gain	761	841	403	352	1.00 (reference)	1.21 (1.01, 1.45)	
	Breastfeeding status	Never	612	633	311	273	1.00 (reference)	1.16 (0.95, 1.43)	0.9
		Ever	307	365	135	135	1.00 (reference)	1.13 (0.85, 1.51)	
	Menopausal status	Pre-	308	328	100	102	1.00 (reference)	0.97 (0.70, 1.35)	0.3
		Post-	592	633	339	292	1.00 (reference)	1.24 (1.02, 1.51)	

^a^ Earliest self-reported fogger truck at residence stratified by time period among Long Island, New York women, Long Island Breast Cancer Study Project,1996-1997. Population and modifier definitions are mutually exclusive categories.

^b^adjusted for 5-year age groups.

^c^multiplicative interaction p-value from likelihood ratio test.

Study participants were categorized by their birth cohorts, defined based on important periods of DDT use (Table 6). An increased risk of breast cancer (OR = 1.29; 95% CI: 1.10, 1.52) was observed for women who were born between 1925 and 1945 and who reported seeing a fogger truck compared to women in the same birth cohort who did not see a fogger truck. There was a suggestion of an increased in breast cancer risk for seeing a fogger truck for women born before 1925 (OR = 1.35; 95% CI: 0.95, 1.89). No increase in risk was observed for women born after 1945.

**Table 6:** **Association between fogger truck and breast cancer risk, stratified by birth cohort**^**a**^

**Birth cohort**	**Population**	**Self-reported fogger truck at residence**	**Cases N (%)**	**Controls N (%)**	**Age-adjusted OR (95% CI)**^**b**^
<1926	Total Population	No fogger	167 (63.0)	138 (69.0)	1.00 reference
		Fogger	98 (37.0)	62 (31.0)	1.28 (0.86, 1.89)
	Fogger truck ≤1972	No fogger	167 (66.5)	138 (72.6)	1.00 reference
		Fogger	84 (33.5)	52 (27.4)	1.35 (0.95, 1.89)
1926-1945	Total Population	No fogger	434 (55.6)	509 (63.1)	1.00 reference
		Fogger	346 (44.4)	298 (36.9)	1.36 (1.11, 1.66)
	Fogger truck ≤1972	No fogger	434 (61.0)	509 (67.7)	1.00 reference
		Fogger	277 (39.0)	243 (32.2)	1.29 (1.10, 1.52)
1946-1972	Total Population	No fogger	318 (68.7)	348 (63.9)	1.00 reference
		Fogger	145 (31.3)	197 (36.2)	0.80 (0.61, 1.04)
	Fogger truck ≤1972	No fogger	318 (78.9)	348 (75.5)	1.00 reference
		Fogger	85 (21.1)	113 (24.5)	0.87 (0.72, 1.04)

^a^Earliest self-reported fogger truck at residence stratified by time period among Long Island, New York women, Long Island Breast Cancer Study Project, 1996-1997. Birth cohorts and population definitions are mutually exclusive categories.

^b^adjusted for 5-year age groups.

The kappa statistic between self-report of ever seeing a fogger truck and lipid-adjusted blood serum concentrations of *p-p’-*DDT (k = −0.062) and *p,p’-*DDE (k = −0.060) did not indicate any agreement. Additionally, an adjusted linear regression model to predict log transformed *p-p’-*DDT and *p-p’-*DDE concentrations from self-report of a fogger truck at a residence did not identify an association (Table 7) (*p-p’-*DDT, β = −0.0240, SE = 0.0387; *p-p’-* DDE, β = −0.1002, SE = 0.0569; *p-p’-*DDT/ *p-p’-*DDE, β = 1.5783, SE = 4.6883).

**Table 7:** **Prediction of *p,p’-*DDT, *p,p’*-DDE and *p,p’*-DDT */ p,p’ *-DDE ratio serum concentrations by seeing a fogger truck**^**a**^

**Population**	**β**	**ln DDT**^**b**^	**ln DDE**^**b**^	**ln (DDT/DDE)**^**b**^
		**n = 1,032**	**n = 1,051**	**n = 1,032**
Total LIBCSP population	β	−0.0228	−0.0854	−0.4080
	SE	0.0378	0.0542	4.6704
	p^c^	0.5	0.1	0.9
Fogger truck ≤1972	β	−0.0240	−0.1002	1.5783
	SE	0.0387	0.0569	4.6883
	p^c^	0.5	0.08	0.7

^a^ Earliest self-reported fogger truck at residence stratified by time period among Long Island, New York women, Long Island Breast Cancer Study Project,1996-1997. Population definitions are mutually exclusive categories.

^b^Adjusted for 5-year age groups, race (white, other), BMI at reference (CDC categories), weight change from age 20 to diagnosis (lost, stable, gained), breastfeeding (ever/never), lipid levels, cholesterol(g/L) and trigylcerides(g/L).

^c^Wald χ^2^ p-value.

## Discussion

This is the first study to consider whether there is an association between reports of seeing a fogger truck and the risk of breast cancer. Among the LIBCSP population as a whole, there is little evidence that ever seeing a fogger truck was associated with an increase in risk of breast cancer, however, effect estimates were stronger among certain biologically susceptible subgroups. The strongest, most robust findings were observed for women with hormone receptor positive breast cancer.

Individuals with ER+PR+ tumors were 44% more likely to have reported ever seeing a fogger truck than were participants with other breast cancer subtypes. When restricted to those who were living at the residence ≤14 and ≤20 years of age, women with ER+PR+ tumors were similarly more likely to report seeing a fogger truck than other subtypes. ER+PR+ tumors, which include Luminal A and Luminal B tumors [5], are driven by estrogen and progesterone levels and most commonly associated with reproductive risk factors [8]. This is consistent with xenoestrogenic activity of commercial DDT [39]. Laboratory studies have found that *p-p’-*DDT enhances ER+PR+ tumor growth [40,41]. Therefore, it would be expected to observe the strongest effect of DDT exposure among women with this hormonally active subtype. A stronger risk of ER+PR+ tumors was not found in a previous LIBCSP report that focused on *p-p’-*DDT and *p-p’-* DDE serum concentrations, but we did not consider self-reported exposure to fogger trucks in that analysis [17]. ER+PR+ are the most common subtype of breast cancer in American women with approximately 70% of tumors classified as ER+PR+ or “hormonally responsive” [42]. The high prevalence of ER+PR+ breast cancer underscores the need to better understand the etiology of these specific tumors in an effort to improve public health risk reduction strategies in the U.S.

When stratifying by birth cohort, we found that seeing a fogger truck was most detrimental among women who were 20 or younger at the time DDT was widely introduced in the U.S in 1945. It is plausible that any effect of seeing a fogger truck after those early years was masked by the ubiquitous exposure to DDT. This result suggests that seeing a fogger truck had the most detrimental effect before exposure became omnipresent. The increased risk observed for postmenopausal women is likely due to the fact that women diagnosed with premenopausal breast cancer in 1996–1997 were less likely to be born in the earlier birth cohorts where an effect of DDT on breast cancer risk was observed.

The proxy measure of a fogger truck used in this study suggests that *p,p’*-DDT, rather than the metabolite *p,p’*-DDE, may be the carcinogenic compound; a result that is consistent with laboratory evidence and the Cohn *et al.,* study [12,24]. This finding may also help to explain discrepancies in the previous literature that often relied on DDE measurements or did not investigate the association by tumor subtype.

Although the heterogeneity in the ORs for the associations between seeing a fogger truck and breast cancer by change in weight was not significant, the study power for evaluating the interaction was low because of the small number of LIBCSP participants who maintained or lost weight. The elevated risk of breast cancer among those who gained weight and reported seeing a fogger truck compared to those who did not see a fogger truck at their residence suggests that DDT may have a stronger or more detectable effect when stored in adipose tissue of women who gain and store weight over time.

There was not a large difference in the results when >1972 reports of exposure to fogger trucks were excluded. However, due to the possible use of other pesticides after 1972 by fogger trucks it was important to consider possible exposure misclassification and to better isolate the effects of DDT exposure.

*p-p’-*DDT and *p-p’-*DDE serum concentrations, a surrogate measure of long-term exposures, were not correlated with seeing a fogger truck at a residence, a surrogate measure of acute exposures. This result is not unexpected given the long period from exposure to blood collection in this study, and evidence that current organochlorine serum concentrations may be decoupled from exposure levels due to intakes from food sources [43] and differing metabolism and degree of organochlorine storage in individuals [44]. As well, this measure of acute exposure is unlikely to be indicative of total DDT exposure levels as women were likely exposed to commercial DDT by sources outside their residences. This lack of correlation between serum concentrations and the fogger truck measure at least partially explains the discrepancies between the association found here and the previous LIBCSP publication focused on serum concentrations, which did not include the fogger truck exposure [17].

There was no increase in risk observed for reporting a fogger truck at multiple residences. Although the report of seeing a fogger truck may represent a single exposure, it is also possible that spraying occurred repeatedly at the residence which would make it difficult to discern exact exposure levels based on the questionnaire.

There was little evidence of an increased breast cancer risk for seeing a fogger truck with either of the two biologically susceptible windows investigated in this analysis, ≤14 and ≤20 years of age. Although cell sizes were small, slightly stronger estimates were observed among participants who reported seeing a fogger truck at their residence when they were 15–20 years of age.

The LIBCSP has extensive residential history information that allowed for the investigation of exposure during susceptible windows. A strength of this investigation is that the study cohort was alive during DDT use in the U.S., and was exposed at a range of ages. However, despite the relatively large, population-based sample size available, a larger sample would have provided a better opportunity to thoroughly investigate the subgroup analysis of biological windows of susceptibility. Additionally, due to the age of the study population, there were few premenopausal women who were within the hypothesized susceptible windows during the time periods where there was an effect observed for fogger truck exposure. Due to the case–control design of the study, we were not able to completely address age-period-cohort effects that would result in a more definitive case for critical windows of exposure. We were also unable to investigate any impact time spent outdoors or use of local produce that could have contributed to the understanding of this relationship.

Another potential limitation is differential recall bias. Cases who were concerned about their previous exposures to potentially harmful chemicals may have been more likely to report seeing a fogger truck. It is unlikely that this bias would differ by ER/PR status, yet it was when we assessed for potential heterogeneity among these subgroups that one of our strongest findings was observed. Approximately equal proportions of cases and controls reported that they believed that at least one environmental factor could cause breast cancer (69 percent and 68 percent, respectively) [45]; suggesting recall bias was not differential with respect to case–control status. It is difficult to hypothesize at what age seeing a fogger truck could be considered a memorable experience and likely to vary across individuals. Any variation in recall would not be expected to differ by case status and thus would bias estimates towards the null. To the best of our knowledge, the use of any other pesticides in fogger trucks before the DDT ban is unknown and any exposure misclassification resulting from the use of alternative pesticides would attenuate any effect on breast cancer risk, which we assume is attributable to DDT. Alternatively, it is also possible that our results could be due to an unknown chemical or chemicals and not attributable to DDT.

We did not observe significant heterogeneity when investigating breast cancer risk by birth cohort (data not shown) and therefore it is unlikely that truck sightings are proxies for age at diagnosis. From the lack of an increase in risk observed for seeing a truck among the 1946–1972 birth cohort, we can also conclude that seeing a fogger truck was not a proxy for being at a young and potentially susceptible age during DDT use.

Few studies have examined the association between DDT exposure during younger ages and subsequent breast cancer risk, and none have considered acute DDT exposures. Therefore, the study results reported here provide a novel perspective to the ongoing discussion about the relationship between DDT and breast cancer. The stronger association observed with a biologically susceptible subgroup, namely women with ER+PR+ breast cancer, provide support for an association between DDT and breast cancer risk. With the prevalence of DDT increasing with indoor residual spraying programs for malaria vector control outside the U.S. [46], the importance of understanding the health effects of DDT continues.

## Conclusions

In this first report that considers acute exposure to DDT, a positive association was observed for spraying fogger trucks and ER+PR+ breast cancer. Given the high incidence of breast cancer, and in particular ER+PR+ tumors [42], this finding suggests that increased risks for tumor subgroups may have significant public health importance in areas where DDT continues to be used. Although the potential association between DDT and breast cancer has been extensively addressed in previous studies, results are inconsistent [9]. The novel approach undertaken here, which focuses on the associations with seeing a fogger truck as a proxy for acute DDT exposure, provides new information regarding the relationship with ER+PR+ breast cancer subtype. Therefore, these findings, based on consideration of exposure timing and breast cancer subtype, may help to guide future investigations on the potential impact and carcinogenic mechanisms of DDT and breast cancer.

## Abbreviations

ER+PR+: Estrogen receptor/ progesterone receptor-positive; p,p’-DDT: *p,p’-*dichlorodiphenyltrichloroethane; p,p’-DDE: *p,p’-*dichlorodiphenyldichloroethylene; EPA: U.S. Environmental Protection Agency; LIBCSP: Long Island Breast Cancer Study Project

## Competing interests

The authors declared that they have no competing interests.

## Authors’ contributions

AW, ST and MG conceived and designed the analysis. AW and MG drafted the manuscript. AW carried out the statistical analysis. MW completed the organochlorine assays. MG was Principal Investigator, AN was co-Principal Investigator, and ST, MW and SS were collaborators on the LIBCSP, and thus were responsible for all data collection (study design and planning, including subject identification, recruitment, interview administration and phlebotomy). All authors contributed to the revisions of the manuscript and read and approved the final manuscript.

## Supplementary Material

Additional file 1 Breast cancer subtype, stratified by birth cohorts, LIBCSP, 1996-1997. Click here for file

## References

[B1] American Cancer Society Cancer Facts and Figures 2011 2011 Atlanta, GA: American Cancer Society

[B2] Yasui Y Potter JD The shape of age-incidence curves of female breast cancer by hormone-receptor status Cancer Causes Control 1999 10 431 437 10.1023/A:1008970121595 10530614

[B3] Carey LA Perou CM Livasy CA Dressler LG Cowan D Conway K Karaca G Troester MA Tse CK Edmiston S Deming SL Geradts J Cheang MC Nielsen TO Moorman PG Earp HS Millikan RC Race, breast cancer subtypes, and survival in the Carolina Breast Cancer Study JAMA 2006 295 2492 2502 10.1001/jama.295.21.2492 16757721

[B4] Ma H Wang Y Sullivan-Halley J Weiss L Marchbanks PA Spirtas R Ursin G Burkman RT Simon MS Malone KE Strom BL McDonald JA Press MF Bernstein L Use of four biomarkers to evaluate the risk of breast cancer subtypes in the women’s contraceptive and reproductive experiences study Cancer Res 2010 70 575 587 10.1158/0008-5472.CAN-09-3460 20068186 PMC2807992

[B5] Muss HB Coming of age: breast cancer in seniors Oncologist 2010 15 Suppl 5 57 65 21138956 10.1634/theoncologist.2010-S5-57

[B6] Phipps AI Malone KE Porter PL Daling JR Li CI Reproductive and hormonal risk factors for postmenopausal luminal, HER-2-overexpressing, and triple-negative breast cancer Cancer 2008 113 1521 1526 10.1002/cncr.23786 18726992 PMC2587413

[B7] Yang XR Chang-Claude J Goode EL Yang XR Chang-Claude J Goode EL Couch FJ Nevanlinna H Milne RL Gaudet M Schmidt MK Broeks A Cox A Fasching PA Hein R Spurdle AB Blows F Driver K Flesch-Janys D Heinz J Sinn P Vrieling A Heikkinen T Aittomaki K Heikkila P Blomqvist C Lissowska J Peplonska B Chanock S Figueroa J Associations of breast cancer risk factors with tumor subtypes: a pooled analysis from the Breast Cancer Association Consortium studies J Natl Cancer Inst 2011 103 250 263 10.1093/jnci/djq526 21191117 PMC3107570

[B8] Yang XR Sherman ME Rimm DL Lissowska J Brinton LA Peplonska B Hewitt SM Anderson WF Szeszenia-Dabrowska N Bardin-Mikolajczak A Zatonski W Cartun R Mandich D Rymkiewicz G Ligaj M Lukaszek S Kordek R Garcia-Closas M Differences in risk factors for breast cancer molecular subtypes in a population-based study Cancer Epidemiol Biomarkers Prev 2007 16 439 443 10.1158/1055-9965.EPI-06-0806 17372238

[B9] Lopez-Cervantes M Torres-Sanchez L Tobias A Lopez-Carrillo L Dichlorodiphenyldichloroethane burden and breast cancer risk: a meta-analysis of the epidemiologic evidence Environ Health Perspect 2004 112 207 214 14754575 10.1289/ehp.112-1241830 PMC1241830

[B10] U.S. Environmental Protection Agency (EPA) DDT, a Review of Scientific and Economic Aspects of the Decision to Ban its Use as a Pesticide 1975 Washington, DC: U.S. Environmental Protection Agency

[B11] Wolff MS Toniolo PG Environmental Organochlorine exposure as a potential etiologic factor in breast cancer Environ Health Perspect 1995 103 Suppl 7 141 145 10.1289/ehp.95103s7141 8593861 PMC1518872

[B12] International Agency for Research on Cancer (IARC) DDT and associated compounds 1991 Lyon, France: IARC: Monographs on Evaluation of Carcinogenic Risk to Humans PMC7681469 1683674

[B13] Brown NM Lamartiniere CA Xenoestrogens alter mammary gland differentiation and cell proliferation in the rat Environ Health Perspect 1995 103 708 713 7588483 10.1289/ehp.95103708 PMC1522196

[B14] Snedeker SM Pesticides and breast cancer risk: a review of DDT, DDE, and dieldrin Environ Health Perspect 2001 109 Suppl 1 35 47 11250804 10.1289/ehp.01109s135 PMC1240541

[B15] Bouwman H van den Berg H Kylin H DDT and malaria prevention: addressing the paradox Environ Health Perspect 2011 119 744 747 10.1289/ehp.1002127 21245017 PMC3114806

[B16] Wolff MS Toniolo PG Lee EW Rivera M Dubin N Blood levels of organochlorine residues and risk of breast cancer J Natl Cancer Inst 1993 85 648 652 10.1093/jnci/85.8.648 8468722

[B17] Gammon MD Wolff MS Neugut AI Eng SM Teitelbaum SL Britton JA Terry MB Levin B Stellman SD Kabat GC Hatch M Senie R Berkowitz G Bradlow HL Garbowski G Maffeo C Montalvan P Kemeny M Citron M Schnabel F Schuss A Hajdu S Vinceguerra V Niguidula N Ireland K Santella RM Environmental toxins and breast cancer on Long Island. II. Organochlorine compound levels in blood Cancer Epidemiol Biomarkers Prev 2002 11 686 697 12163320

[B18] Brody JG Aschengrau A McKelvey W Rudel RA Swartz CH Kennedy T Breast cancer risk and historical exposure to pesticides from wide-area applications assessed with GIS Environ Health Perspect 2004 112 889 897 10.1289/ehp.6845 15175178 PMC1242018

[B19] Charlier C Foidart JM Pitance F Herman P Gaspard U Meurisse M Plomteux G Environmental dichlorodiphenyltrichlorethane or hexachlorobenzene exposure and breast cancer: is there a risk? Clin Chem Lab Med 2004 42 222 227 15061365 10.1515/CCLM.2004.040

[B20] Rubin CH Lanier A Kieszak S Brock JW Koller KR Strosnider H Needham L Zahm S Harpster A Breast cancer among Alaska Native women potentially exposed to environmental organochlorine chemicals Int J Circumpolar Health 2006 65 18 27 16544644 10.3402/ijch.v65i1.17885

[B21] Gatto NM Longnecker MP Press MF Sullivan-Halley J McKean-Cowdin R Bernstein L Serum organochlorines and breast cancer: a case–control study among African-American women Cancer Causes Control 2007 18 29 39 10.1007/s10552-006-0070-2 17186420 PMC1839921

[B22] Iwasaki M Inoue M Sasazuki S Kurahashi N Itoh H Usuda M Tsugane S Japan Public Health Center-based Prospective Study Group Plasma organochlorine levels and subsequent risk of breast cancer among Japanese women: a nested case–control study Sci Total Environ 2008 402 176 183 10.1016/j.scitotenv.2008.05.009 18555519

[B23] Siddiqui MK Anand M Mehrotra PK Sarangi R Mathur N Biomonitoring of organochlorines in women with benign and malignant breast disease Environ Res 2005 98 250 257 10.1016/j.envres.2004.07.015 15820732

[B24] Cohn BA Wolff MS Cirillo PM Sholtz RI DDT and breast cancer in young women: new data on the significance of age at exposure Environ Health Perspect 2007 115 1406 1414 17938728 10.1289/ehp.10260 PMC2022666

[B25] Hiatt RA Haslam SZ Osuch J Breast Cancer and the Environment Research Centers The breast cancer and the environment research centers: transdisciplinary research on the role of the environment in breast cancer etiology Environ Health Perspect 2009 117 1814 1822 20049199 10.1289/ehp.0800120 PMC2799453

[B26] Cohn BA Developmental and environmental origins of breast cancer: DDT as a case study Reprod Toxicol 2011 31 302 311 10.1016/j.reprotox.2010.10.004 20965245 PMC3268657

[B27] Longnecker MP Invited Commentary: Why DDT matters now Am J Epidemiol 2005 162 726 728 10.1093/aje/kwi277 16120697

[B28] Wolff MS Berkowitz GS Brower S Senie R Bleiweiss IJ Tartter P Pace B Roy N Wallenstein S Weston A Organochlorine exposures and breast cancer risk in New York City women Environ Res 2000 84 151 161 10.1006/enrs.2000.4075 11068929

[B29] Colditz GA Frazier AL Models of breast cancer show that risk is set by events of early life: prevention efforts must shift focus Cancer Epidemiol Biomarkers Prev 1995 4 567 571 7549816

[B30] John EM Kelsey JL Radiation and other environmental exposures and breast cancer Epidemiol Rev 1993 15 157 162 8405198 10.1093/oxfordjournals.epirev.a036099

[B31] Gammon MD Neugut AI Santella RM Teitelbaum SL Britton JA Terry MB Eng SM Wolff MS Stellman SD Kabat GC Levin B Bradlow HL Hatch M Beyea J Camann D Trent M Senie RT Garbowski GC Maffeo C Montalvan P Berkowitz GS Kemeny M Citron M Schnabe F Schuss A Hajdu S Vincguerra V Collman GW Obrams GI The Long Island Breast Cancer Study Project: description of a multi-institutional collaboration to identify environmental risk factors for breast cancer Breast Cancer Res Treat 2002 74 235 254 10.1023/A:1016387020854 12206514

[B32] Brock JW Burse VW Ashley DL Najam AR Green VE Korver MP Powell MK Hodge CC Needham LL An improved analysis for chlorinated pesticides and polychlorinated biphenyls (PCBs) in human and bovine sera using solid-phase extraction J Anal Toxicol 1996 20 528 536 10.1093/jat/20.7.528 8934301

[B33] Selvin S Statistical Analysis of Epidemiologic Data 1996 Second New York: Oxford University Press

[B34] Shrier I Platt RW Reducing bias through directed acyclic graphs BMC Med Res Methodol 2008 8 70 10.1186/1471-2288-8-70 18973665 PMC2601045

[B35] Hosmer D Lemeshow S Applied Logsitic Regression 1989 New York: John Wiley & Sons

[B36] Stevens J Truesdale KP McClain JE Cai J The definition of weight maintenance Int J Obes (Lond) 2006 30 391 399 10.1038/sj.ijo.0803175 16302013

[B37] Cohen J A coefficient of agreement for nominal scales Educ Psychol Meas 1960 20 37 46 10.1177/001316446002000104

[B38] Phillips DL Pirkle JL Burse VW Bernert JT Jr Henderson LO Needham LL Chlorinated hydrocarbon levels in human serum: effects of fasting and feeding Arch Environ Contam Toxicol 1989 18 495 500 10.1007/BF01055015 2505694

[B39] Shekhar PV Werdell J Basrur VS Environmental estrogen stimulation of growth and estrogen receptor function in preneoplastic and cancerous human breast cell lines J Natl Cancer Inst 1997 89 1774 1782 10.1093/jnci/89.23.1774 9392618

[B40] Robison AK Sirbasku DA Stancel GM DDT supports the growth of an estrogen-responsive tumor Toxicol Lett 1985 27 109 113 10.1016/0378-4274(85)90127-4 4060181

[B41] Scribner JD Mottet NK DDT acceleration of mammary gland tumors induced in the male Sprague–Dawley rat by 2-acetamidophenanthrene Carcinogenesis 1981 2 1235 1239 10.1093/carcin/2.12.1235 7326823

[B42] Potter JD Cerhan JR Sellers TA McGovern PG Drinkard C Kushi LR Folsom AR Progesterone and estrogen receptors and mammary neoplasia in the Iowa Women’s Health Study: how many kinds of breast cancer are there? Cancer Epidemiol Biomarkers Prev 1995 4 319 326 7655325

[B43] Longnecker MP Rogan WJ Lucier G The human health effects of DDT (dichlorodiphenyltrichloroethane) and PCBS (polychlorinated biphenyls) and an overview of organochlorines in public health Annu Rev Public Health 1997 18 211 244 10.1146/annurev.publhealth.18.1.211 9143718

[B44] Sholtz RI McLaughlin KR Cirillo PM Petreas M Park JS Wolff MS Factor-Litvak P Eskenazi B Krigbaum N Cohn BA Assaying organochlorines in archived serum for a large, long-term cohort: implications of combining assay results from multiple laboratories over time Environ Int 2011 37 709 714 10.1016/j.envint.2011.01.013 21333355 PMC3257216

[B45] Teitelbaum SL Gammon MD Britton JA Neugut AI Levin B Stellman SD Reported residential pesticide use and breast cancer risk on Long Island New York. Am J Epidemiol 2007 165 643 651 10.1093/aje/kwk046 17166928

[B46] Van den Berg H Global status of DDT and its alternatives for use in vector control to prevent disease Environ Health Perspect 2009 117 1656 1663 10.1289/ehp.0900785 20049114 PMC2801202

