# Supplementary material for: Exposure to fogger trucks and breast cancer incidence in the Long Island Breast Cancer Study Project: a case–control study
Source: Environ Health. 2013 Mar 15;12:24. doi: 10.1186/1476-069X-12-24 (PMC3639887; doi:10.1186/1476-069X-12-24)
Supplement: Additional file 1 — Breast cancer subtype, stratified by birth cohorts, LIBCSP, 1996-1997. [file 1476-069X-12-24-S1.doc]

**Supplemental Table I. Breast cancer subtype, stratified by birth cohorts, LIBCSP, 1996-1997.**

|  | ER-PR-  N (%) | Other Subtypes^a^  N(%) | ER+PR+  N(%) | Other Subtypes^b^  N(%) |
| --- | --- | --- | --- | --- |
| <1925 | 31 (14.6) | 173 (22.2) | 125 (21.4) | 79 (19.4) |
| 1925-1945 | 102 (48.1) | 392 (50.4) | 287 (49.3) | 207 (50.9) |
| 1946-1972 | 79 (37.2) | 213 (27.4) | 171 (29.3) | 121 (29.7) |
| >1972 | 0 (0.0) | 0 (0.0) | 0 (0.0) | 0 (0.0) |

^a^ For ER-PR- vs. Other Subtypes comparison, “Other Subtypes” includes ER+PR-, ER+PR+ and ER-PR+

^b^ For ER+PR+ vs Other Subtypes comparison, “Other Subtypes” includes ER+PR-, ER-PR- and ER-PR+.
